# Qualitative and Quantitative Evaluation of Donor Corneal Tissue by Slit Lamp and Specular Microscopy

**DOI:** 10.7759/cureus.24700

**Published:** 2022-05-03

**Authors:** Mayur Patil, Abhay Lune, Radhika Paranjape, Kunj Naik, Vishakh Padmakumar, Aparna Alapati, Divya Motwani, Amod Ahuja, Nilay Dhore, Sucheta Kaul

**Affiliations:** 1 Ophthalmology, Dr. D. Y. Patil Medical College, Hospital & Research Centre, Pune, IND; 2 Ophthalmology: Pediatrics, L V Prasad Eye Institute, Hyderabad, IND; 3 Ophthalmology, L V Prasad Eye Institute, Hyderabad, IND

**Keywords:** hexagonality, coefficient of variation, endothelial cell density, donor cornea, corneal blindness

## Abstract

Background

In India, donor eye collection and promotion of eye banking are insufficient to meet the needs. By adequately evaluating donor corneas, eye banks can maximize the number of viable corneas for transplantation. This study evaluated donor corneal tissue based on age, lens status, and cause of death by their morphology and endothelial cell count via slit lamp and specular microscopy.

Methods

We conducted a prospective observational study of all eye bank donor corneas indicated for eye donation at a tertiary hospital and research center in Western Maharashtra between September 2019 to December 2021. We evaluated the corneoscleral discs by slit-lamp microscopy specular microscopy. We analyzed donor corneas quantitatively and qualitatively and graded them accordingly. We also collected blood samples for serological testing and the donor's behavioral and family medical histories.

Results

We collected 94 eyes from 47 donors; the mean age of the donor population was 48.2 years, and most donors were aged 41 to 80 years. Thirty-one donors (65.96%) were male, and 16 were female (34.04%. For preservation, we used Cornisol (Aurolab, Madurai, India) in 36 cases (77%) and McCarey-Kaufman medium in 11 cases (23%). We found a mean endothelial cell density (ECD) of 2214.40/mm2, with hexagonality of 53.05%, and a coefficient of variation of 38.01. Further, we observed that ECD and hexagonality of cells in phakic donors were significantly greater than that of pseudophakic (PP) donors. Moreover, ECD and hexagonality significantly decreased in donors with the chronic disease compared to those who had a sudden, unexpected death.

Conclusion

Corneal grafts from younger donors, phakic donors, and donors who experienced an acute cause of death were qualitatively and quantitatively significantly better than those of older donors, PP donors, and donors who experienced sudden, unexpected death. Therefore, eye bank specular examination can improve tissue utilization and transplantation success. Therefore, we strongly recommend that eye bank personnel evaluate their donor tissue with a specular microscope to enhance the quality of eye care.

## Introduction

Reduced vision or blindness due to cornea diseases is known as corneal blindness. In most cases (95%), corneal blindness can be prevented with early diagnosis and appropriate treatment of corneal ulceration using suitable antibiotics and antifungal medications [1,2]. Further, corneal transplants can restore vision to the recipients in most cases [3]. According to a World Health Organization survey, one person goes blind every five seconds [4], and only one donor corneal tissue is available for every 70 patients requiring corneal tissue [5].

Furthermore, according to a national program for the control of blindness census, there are 120,000 corneal blind patients in India, with 25,000 to 30,000 new patients each year [1]. By 2020, India's number of people with unilateral corneal blindness will rise to 10.6 million [1]. Corneal blindness is the second most cause of preventable and treatable blindness in our country [6]. Approximately 22,000 eyes are collected every year in India, which is much less than the amount needed [7]. Therefore, there is a need to promote eye donation and improve the evaluation of donor corneas to improve their utilization rate. The endothelial cells can be precisely evaluated using a specular microscope, and they may get deemed suitable for transplantation, which can improve their utilization rates. In recent years, significant progress has been achieved in corneal procurement, preparation, and processing, resulting in a significant increase in the number and quality of eligible corneas for transplant [7,8].

The success of corneal transplantation largely depends on the quality of the donor cornea, which is determined by a thorough examination. The donor's cause of death, ocular state, and the period from death to enucleation/retrieval are essential factors. Gross inspection, qualitative slit-lamp biomicroscopy, and quantitative specular microscopy should be done to evaluate the transplant suitability of the donor cornea [9-11]. Therefore, this study aimed to evaluate donor corneal tissue according to age, lens status, cause of death, morphology, and endothelial cell count via slit lamp and specular microscopy.

## Materials and methods

We conducted a prospective observational study of donors' eyes at a tertiary hospital and research center in Western Maharashtra between September 2019 to December 2021. The Institutional Ethics Subcommittee approved the study (Research Protocol No. IESC/PGS/2019/114). All eye bank donor corneas indicated for eye donation during the study period were included, and family/next of kin provided written informed consent prior to cornea collection.

Corneas were collected within six hours of the time of death. All the procedures were conducted in aseptic conditions. We excised the donor corneoscleral disc in situ and stored the tissue immediately in McCarey Kaufman (MK) or Cornisol (Aurolab, Madurai, India) preservative media at 4°C. Blood samples were collected for serological testing. We also recorded the donor's behavioral history and family medical history.

We used narrow and diffuse slit-lamp microscopy to examine the epithelium for integrity and overall condition, specifically for exposure keratopathy, sloughing, abrasions, defects, and foreign bodies. The stroma was examined for overall clarity, opacity, amount of edema, and folding of Descemet's membrane (DM). We used the retro illumination light technique to assess the endothelial layer for stress line, guttate, iris pigments on endothelium, endothelial defect, and peels. A clinical-grade was assigned to the corneas ranging from excellent to poor, according to Saini et al.'s criteria [9].

To determine endothelial cell count and morphology of the donor corneas, we used specular microscopy and the CellChek D eye bank keratoanalyzer (Konan Medical USA, Inc., Irvine, CA). The sample was analyzed after bringing the preservative container temperature to approximately 18°C to 22°C. We used the center method with fixed frame analysis to obtain the endothelial cell density (ECD), coefficient of variation (CV), and percentage of hexagonality. This procedure was repeated for each cornea for four frames, with 90 to 100 cells selected and counted from each frame [11]. We analyzed the donor corneas quantitatively and qualitatively and graded them as therapeutic or optical grade corneas. Specular microscopy grading was done according to Chaurasia et al. [12].

Statistical analysis

The data collected from the donor records were tabulated and analyzed using IBM SPSS Statistics for Windows, Version 22.0. (IBM Corp., Armonk, NY). We used paired and unpaired t-tests to compare the variables in the groups, and we considered p≤0.05 as statistically significant.

## Results

A total of 94 donor eyes from 47 patients were examined in this investigation. The donor population's mean age was 48.2 years with a standard deviation of 48.2±13.77, with most donors aged 41 to 80 years. One donor (2%) was older than age 80, while two donors (4%) were younger than age 20, and 10 donors (26%) were ages 21 to 40 years. In most cases, the donor's eyes were obtained within six hours of death. MK media was used to store 22 eyes from 11 donors, while Cornisol media was used to store 72 eyes from 36 donors. They were then stored at 4°C to 8°C until further processing.

Slit-lamp microscopy was used to analyze the epithelium, stroma, Descemet's membrane, and endothelium. The epithelium was intact in 70 eyes (70.4%), and keratopathy was observed in 14 eyes (14.9%), followed by an epithelial defect in five eyes (5.3%). We noted sloughing, debris, and mild exposure in five eyes (5.3%). Compact stroma was observed in 75 eyes (79.7%), followed by haze, mild edema, and thick arcus in eight (8.5%), six (6.3%), and five (5.3%) eyes respectively. Further, no DM folds were observed in 61 eyes (64.9%), whereas Grade 1 and Grade 2 folds were observed in 20 (21.2%) and 13 (13.8%) eyes, respectively. Endothelium was normal in 79 eyes (84.0%), whereas guttata could be seen in 11 eyes (11.7%). Stress lines were visible in four eyes (4.2%).

**Figure 1:**
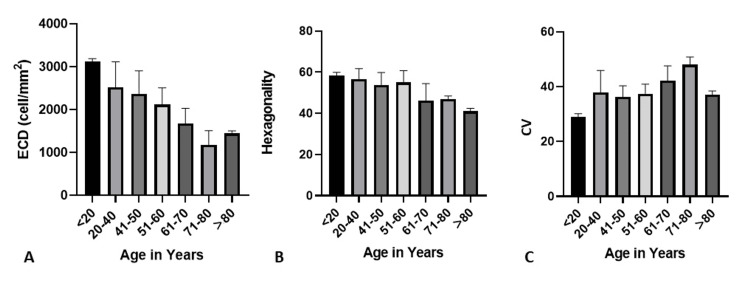
ECD (A), Hexagonality (B), and CV (C) according to donor age ECD, endothelial cell density; CV, coefficient of variance.

We observed a significant and steady decline in ECD with age. For donors younger than age 20, ECD was 3125 ± 64.55 cells/mm2, which reduced 1175±332.34 cells/mm2 for donors aged 71 to 80 (Table 1; Figure 1A). The mean hexagonality of cells also showed a decreasing trend with age. However, no significant difference was observed between those donors younger than 20 to age 60. Mean hexagonality further showed a significant decline in donors aged 60 to older than 80 (Figure 1B). The mean CV increased with age, displaying an inverse relationship with hexagonality. Mean CV significantly increased from 29 ± 1.15 for donors younger than 20 years to 37.92 ± 7.95 for donors aged 20 to 40 years. However, a significant increase was not seen again afterward until donors were aged 60 or older (Figure 1C).

**Table 1:** Specular microscopy examination of donor's eye CV, coefficient of variation; ECD, endothelial cell density; SD, standard deviation; *, statistically significant

Age range (Years)	Donors, n (%)	ECD (cells/mm^2^), Mean ± SD	P-Value	Hexagonality (Mean ± SD)	P-Value	CV (Mean ± SD)	P-Value
< 20	2 (4)	3125 ± 64.55		58.25 ± 1.70		29 ± 1.15	
21-40	12 (26)	2522.83 ± 591.94	*0.0001	56.66 ± 5.10	0.2589	37.92 ± 7.95	*0.0001
41-50	13 (27.7)	2368.73 ± 537.43	0.3415	53.69 ± 6.16	0.0685	36.31 ± 3.96	0.3778
51-60	9 (19.14)	2114.78 ± 394.94	0.0781	55.06 ± 5.64	0.4526	37.44 ± 3.48	0.3206
61-70	9 (19.14)	1678.33 ± 349.03	*0.0013	46.16 ± 8.26	*0.0007	42.11 ± 5.49	*0.0049
71-80	1 (2)	1175 ± 332.34	0.2501	47 ± 1.41	0.7099	48 ± 2.82	0.1325
>80	1 (2)	1446.5 ± 60.10	0.0693	41 ± 1.41	0.0513	37 ± 1.41	0.0693
Total	47	2214.40 ± 613.82		53.05 ± 7.32		38.0 ± 6.13	

**Figure 2:**
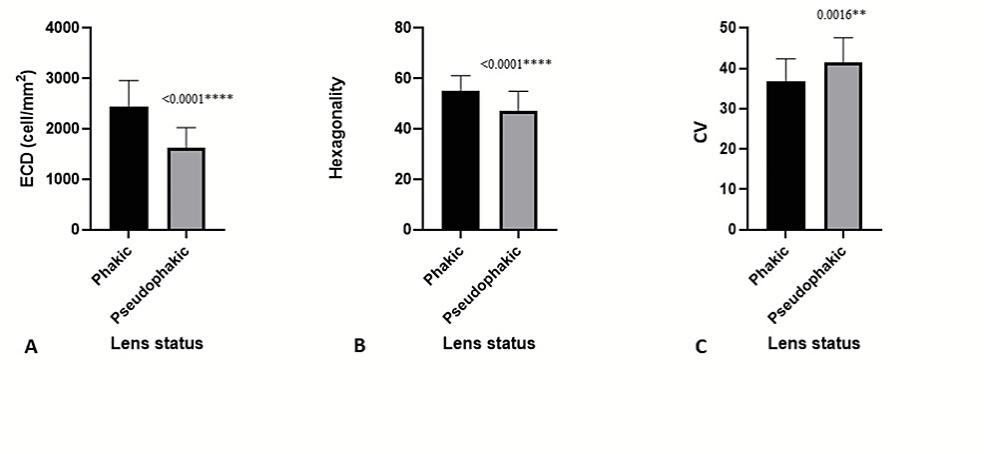
ECD (A), Hexagonality (B), and CV (C) according to phakic or pseudophakic status ECD, endothelial cell density; CV, coefficient of variance.

Further, we classified donor eyes into phakic and pseudophakic (PP) groups and compared them based on specular microscopic examination to check their suitability for transplantation. Of 94 eyes, 69 were phakic, and 25 eyes were PP (Table 2). We observed a significant decrease in the ECD and hexagonality in the PP group compared to the phakic group. The mean CV was significantly higher in PP eyes (41.48 ± 6.18) than in phakic eyes (36.74 ± 5.66; Figure 2).

**Table 2:** Specular microscopy examination according to phakic status CV, coefficient of variation; ECD; endothelial cell density; SD, standard deviation; * statistically significant

Lens Status	Donor eyes, n	ECD (cells/mm^2^), Mean ± SD	P-Value	Hexagonality (Mean ± SD)	P-Value	CV (Mean ± SD)	P-Value
Phakic	69	2430.84 ± 525.84	*0.0001	55.17 ± 5.91	<0.0001	36.74 ± 5.66	0.0016
Pseudophakic	25	1617.04 ± 412.5		47.2 ± 7.75		41.48 ± 6.18	

**Figure 3:**
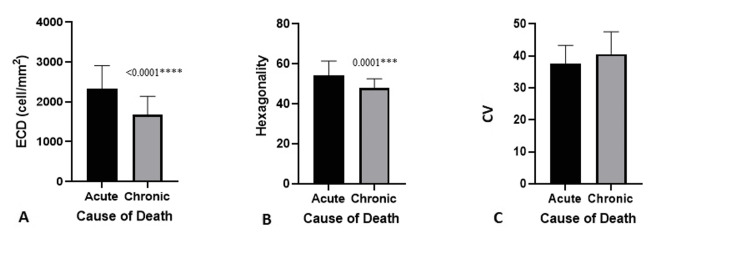
ECD (A), Hexagonality (B), and CV (C) according to cause of death, where acute indicates sudden, unexpected death ECD, endothelial cell density; CV, coefficient of variance.

We also evaluated the impact of the cause of death on ECD, hexagonality, and CV using specular microscopy. Eyes obtained from donors who experienced sudden, unexpected death had better ECD and hexagonality, whereas there was no significant change in the CV (Table 3, Figure 3).

**Table 3:** Specular microscopy observations based on the cause of death CV, coefficient of variation; ECD, endothelial cell density; SD, standard deviation; *, statistically significant

Cause of Death	Donors, n	ECD (cells/mm^2^), Mean ± SD	P-Value	Hexagonality (Mean ± SD)	P-Value	CV (Mean ± SD)	P-Value
Sudden, unexpected	39	2323.54 ± 586.45	*0.0001	54.12 ± 7.33	*0.0001	37.47 ± 5.85	0.1142
Chronic	8	1682.38 ± 454.59	*0.0001	47.88 ± 4.69	*0.0001	40.56 ± 6.99	0.1142

Finally, we used specular microscopy to assign overall grades to the donor corneas. Most corneas were graded as fair (n=24, 25.5%), followed by good and very good (n=25, 26.6% each). Only nine corneas were graded as excellent (9.5%; Table 4).

**Table 4:** Overall grading on corneas based on specular microscopy observation

Overall Corneal Grading	N (%)
Excellent	9 (9.5%)
Very good	25 (26.6%)
Good	25 (26.6%)
Fair	24 (25.5%)
Poor	10 (10.6%)

## Discussion

Our study aimed to evaluate donor cornea tissue by slit-lamp examination and specular microscopy for all corneas retrieved at a tertiary care hospital and research center from September 2019 to December 2021.

The demographics of our study population were similar to several previous studies [13,14]. However, Kapur et al. had a younger mean population age and a female majority of donors like Galgauskas et al. [15,16]. In our study, all corneas were retrieved within six hours of death, complying with India's Joint Review of Eye Banking Standards [17]. Our average death-to-preservation time was three hours, aligning with a previous study [14].

Historically, MK medium is the first successful method for storing excised cornea (corneoscleral button) in a chemically defined tissue culture medium at 4°C for up to four days and has been reported to be efficient [11,15]. However, we preserved most donor tissues (77%) in Cornisol, an intermediate storage medium as effectual as Optisol-GS (Bausch & Lomb Inc., Laval, Canada) in preserving donor corneal tissues for 14 days [14]. Optisol-GS is very effective in preserving ECD and hexagonality but is approximately 10 times the cost of conventional MK media [15]. Therefore, in low-income countries like India, eye banks continue to place corneal tissue in MK media initially, as it can keep tissue viable for up to four days. However, the need for longer duration storage is rising. There has been an exponential increase in corneal tissue collection by a few eye banks, with more tissue collected than utilized. Better storage options are needed to preserve tissue viability when the tissue must be transported to distant locations [18].

The ECD limit at which endothelium can no longer ensure its normal function is 2,000 cells/mm2. Young and healthy individuals have higher ECD than older people and those with cataracts or glaucoma. Further, the human cornea becomes thinner with age [19,20]. Our ECD, hexagonality, and CV results were similar to those reported by Gupta et al. and Kapur et al. [11,15].

In our study, eyes obtained from donors experiencing sudden, unexpected death had better ECD and hexagonality than those who did not, but we saw no significant difference in the CV. Similar observations were reported in earlier studies that retrieved tissues from road collision-related [9,21,22]. Dasar et al. also reported that long-lasting severe diseases like cancer (which causes cachexia and catabolism) reduce the number of viable endothelial cells more than diseases associated with more rapid death [23].

In our study, young donors had higher ECD and hexagonality, but CV was not correlated to age. While ECD significantly declined across all ages, we found no significant difference in hexagonality from ages 20 to 60 and a significant decline in donors aged 60 to older than 80. Tufekci et al. and Gupta et al. also reported that hexagonality and CV did not change with age [11,24]. Several studies on Indian eye donors reported a similar decline of ECD with age [6,14]. A similar inverse relationship between age and ECD was reported by studies outside India, including studies in Portugal, Denmark, and New Zealand [25,26]. The mean ECD in phakic donors was significantly more significant than the ECD in PP donors, which aligns with Sahoo et al. and Probst et al. [27,28].

In the present study, we observed that combining slit-lamp microscopy and specular microscopy allows for an in-depth review of donor tissue and might help upgrade tissues for transplantation use that would otherwise be discarded. Such evaluations help increase the utility rate of donor corneas. This has been supported by previous studies that revised the grading of the cornea after incorporating specular microscopy for the analysis of donor corneas [29].

Limitations

Our study was limited by its small sample size, which reduced its generalizability to larger populations. Because most of the corneas were procured in-house, we could not conduct a proper comparison between in-house and outsourced tissues (such as those from other hospitals). We also did not incorporate follow-up data after keratoplasty operation to evaluate success or failure rates in corneal transplant recipients.

## Conclusions

Corneal grafts from younger donors were qualitatively and quantitatively significantly better than those of older donors. Further, tissues obtained from donors who experienced sudden cause of death were of higher quality and had better ECD than those who experienced chronic cause of death. We also observed that PP patients had lower ECD than phakic patients. Therefore, eye bank specular examination can improve tissue utilization rates and improve immediate outcomes of surgery. Therefore, we strongly recommend using an eye bank specular microscope in every eye bank to enhance the quality of eye care.
